# Rapid development of seizures and PRES in a COVID-19 patient

**DOI:** 10.1016/j.ebr.2021.100436

**Published:** 2021-03-04

**Authors:** Fabiane Santos de Lima, Sara Klein, Faten El Ammar, Shasha Wu, Sandra Rose, James X. Tao, Naoum P. Issa

**Affiliations:** Adult Epilepsy Center, Department of Neurology, 5841 S. Maryland Ave., MC 2030, University of Chicago, Chicago, IL 60637, United States

**Keywords:** COVID-19, PRES, Posterior reversible encephalopathy syndrome, SARS-CoV-2, Case report

## Abstract

Neurological dysfunction has been noted in up to 36% of patients hospitalized with COVID-19, and a variety of mechanisms of neurological injury are possible. Here we report the rapid development of PRES and acute seizures in a patient with COVID-19 infection and sickle cell disease. The combination of COVID and sickle cell disease may raise the risk of PRES and could contribute to the higher mortality rate of COVID in patients with sickle cell disease.

## Highlights

• Posterior reversible encephalopathy syndrome (PRES) with seizures should be suspected in patients with both COVID-19 and sickle cell disease.
• PRES with seizures might be one reason that patients with sickle cell disease have more severe COVID-19 infections.
• COVID-19, either directly or indirectly, may increase the risk of seizures in susceptible patients.

## 1 Introduction

Neurological dysfunction has been noted in up to 36% of patients hospitalized with COVID-19 [1], and a variety of mechanisms of neurological injury are possible. Here we report the rapid development of acute seizures in a patient with COVID-19 infection.

## 2 Case

The patient is a 43-year-old female with sickle cell disease (SCD) and chronic opioid use who become gradually lethargic over 2 days and was found unresponsive at home. Prior to admission medications included morphine IR 60 mg every 4 hours as needed for pain control, gabapentin 300 mg three times a day, and lisinopril 20 mg daily. On presentation she was hypoxemic (SpO2 92% on 5L of oxygen on nasal cannula) with hyperkalemia (6.3 mmol/L), acute renal dysfunction (Serum Crea 804.6 μmol/L and glomerular filtration rate of 5 mL/min), a hemoglobin of 3.48 mmol/L, and leukocytosis of 23.1 cells/L with 93% neutrophils. Serum cytokine IL 6 was elevated at 77.3 pg/mL (ref range ≤ 1.8 pg/mL). A nasal swab SARS-CoV2 RNA qualitative PCR test was positive.

She had a history of epilepsy present at the age of three months, received phenobarbital until early childhood without a subsequent seizure. An EEG for altered mental status one year prior to presentation showed generalized periodic discharges with triphasic morphology that was interpreted as a metabolic encephalopathy. She did not carry a diagnosis of epilepsy at the time of admission according to the ILAE definition [2].

Her mental status rapidly declined after presentation and she was intubated for airway protection. She was started on emergency dialysis for acute renal failure and hyperkalemia. Abnormal movements were observed, initially deemed to be myoclonic jerks from metabolic derangement. The patient was initiated on levetiracetam 250 mg twice daily, with 250 mg after dialysis as outlined in the dosing guidelines for patients with impaired renal function [3]. Her respiratory and clinical status improved over the next 36 hours, so she was extubated.

**Fig. 1:**
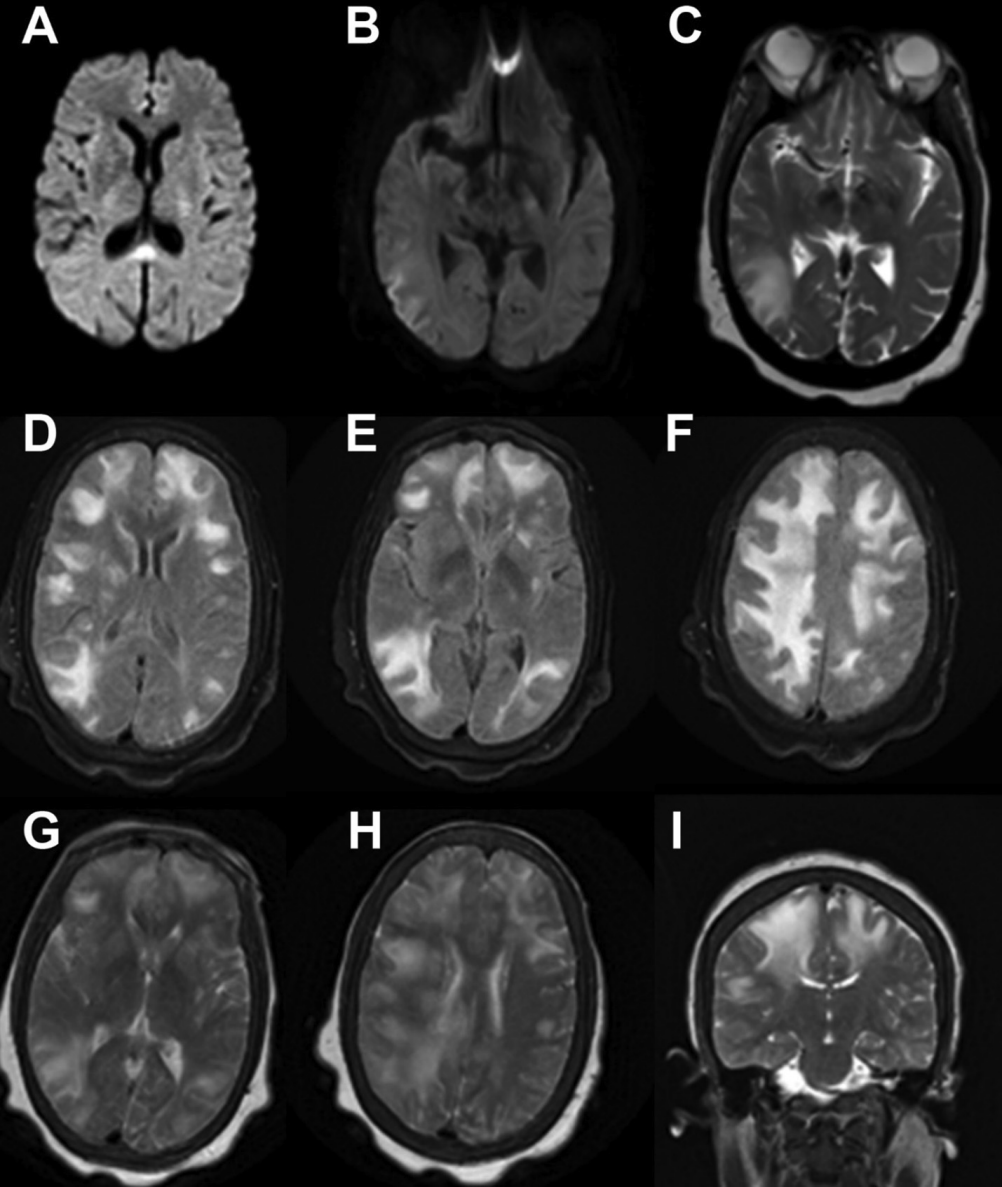
First brain MRI DWI (A) with cytotoxic edema in the splenium of the corpus callosum along with a possible small area of mild cytotoxic edema in the left cerebral white matter Supplementary MRI sequence A. Second brain MRI DWI (B), Axial T2 (C) and Axial Flair (D). Third brain MRI, axial FLAIR hyperintensities (E- I). Supplementary MRI sequence E-I.

However, her mental status remained altered; she was lethargic with periods of wakefulness and only intermittently followed commands. There were no focal findings on her neurological exam. It was not possible to evaluate visual fields. Initial brain magnetic resonance imaging (MRI) showed an area of hyperintesity in the splenium of the corpus callosum, and possibly in the left cerebral white matter (Fig. 1A).

**Fig. 2:**
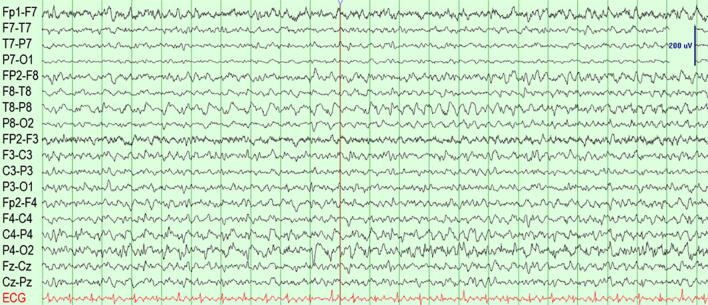
EEG near the onset of a right-temporal seizure. Electrodes are placed in a standard 10–20 international system and displayed in a longitudinal bipolar montage. Vertical lines mark 1-second intervals. The amplitude scale bar shows 200 microvolts.

Within 24 hours she had a convulsive seizure lasting 1–2 minutes and received 2 mg of lorazepam intravenously. An EEG lacked posterior alpha activity and had a dominant background frequency in the delta range with improvement to 5–6 Hz over 144 hours of recording. A total of 54 focal clinical and subclinical seizures were recorded, with onset over the right temporo-parietal region (T8-P8) and propagation to bifrontal regions during some of the seizures (Fig. 2). The semiology of the clinical seizures consisted of left-hand non-clonic rhythmic movement. After the first convulsive seizure, anti-seizure medications were titrated up as renal function improved. Seizures eventually subsided on levetiracetam 750 mg and lacosamide 200 mg twice daily.

Cerebral spinal fluid (CSF) was collected on two occasions during hospitalization, and in neither sample was there sign of CNS infection (Table 1).

**Table 1:** Meningitis/Encephalitis Panel* included Escherichia Coli K1, Haemophilus Influenzae, Listeria Monocytogenes, Neisseria Menigitidis, Streptococcus Agalactiae, Streptococus Pneumoniae, Cytomegalovirus (CMV), Enterovirus, Herpes Simplex Virus 1 (HSV-1), Herpes Simplex Virus 2 (HSV-2), Human Herpes Virus 6 (HHV-6), Human Parechovirus, Varicella Zoster Virus, Cryptococcus Neoformans/Gattii. Encephalopathy-Autoimmune panel** included AMPA-R Antibody CBA, Amphiphysin Antibody, AGNA-1, ANNA-1, ANNA-2, ANNA-3, CASPR2-IgG CBA, CRMP-5-IgG, DPPX Antibody IFA, GABA-B-R Antibody CBA, GAD65 Antibody Assay, GFAP IFA, LGT1-IgG CBA, mGluR1 Antibody IFA, NMDA-R Antibody CBA, PCA-Tr, PCA-1, PCA-2.

CSF findings, reference value	LP 1	LP 2
Appearance, clear	Slightly turbid	Xanthochromic
WBC (cell/µL)	1	3
% Neutrophils, 0%–6%	15	0
% Lymphocytes, 40%–80%	77	83
% Macrophages 15%–45%	8	17
Erythrocytes, 0/μL	684	3028
Glucose, 40–70, mg/dL	69	52
Protein, 15–45, mg/dL	44	94
Meningitis/Encephalitis Panel*	negative	negative
Encephalopathy Autoimmune Panel**	negative	negative
Gram Stain	No organism seen	No organism seen
Bacterial Culture	negative	negative
Fungal Culture	negative	negative
Oligoclonal bands	negative	negative
Cytology	No malignant tumor cells	Not tested

A second brain MRI four days after the first showed extensive areas of T2/FLAIR hyperintensity in the bilateral cerebral hemispheres with mild scattered areas of sulcal enhancement and superimposed gyriform restricted diffusion in the right temporo-occipito-parietal region. The previously observed lesion in the splenium of the corpus callosum had resolved (Fig. 1B, C, D). Another brain MRI three days later showed progression to nearly confluent areas of T2/FLAIR hyperintensity in the right hemispheric white matter and to a lesser extent in the left hemisphere. There was minimal residual diffusion restriction at the right temporo-parietal occipital junction and no clear abnormal enhancement (Fig. 1E, F, G, H, I).

The patient's condition improved over the following four days to her prior baseline, with no focal neurological deficits upon discharge. MRI four months later showed complete resolution of the white matter changes.

## 3 Discussion

This patient had a COVID-19 infection with severe metabolic abnormalities, altered mental status, seizures, and the eventual development then resolution of diffuse T2 hyperintensities on MRI. The differential diagnoses include posterior reversible encephalopathy syndrome (PRES), encephalitis (infectious versus autoimmune), acute disseminated encephalomyelitis (ADEM), cerebral hypoxic injury, and a post-ictal increased T2 signal. The non-inflammatory CSF argues against aseptic (viral) meningitis and suggests the seizures and MRI findings may instead have been a secondary result of SARS-CoV-2 infection rather than direct infection of CNS tissues.

The initial radiographic features including rapid resolution of clinical symptoms and subsequent resolution of MRI abnormalities were most consistent with PRES. PRES can be triggered by a variety of physiological abnormalities, including acute hypertension, renal failure, immunosuppressants, and chemotherapeutics [4]. This patient was mainly normotensive (initially 92–100/49–61; during hospitalization systolic blood pressure ranged from 80 to 160 mm Hg) during her hospital course, requiring short term pressor use at one point, so hypertension would have been an unlikely trigger. She had no history of immunosuppression or recent chemotherapy. On initial presentation, however, she had acute renal failure requiring a short course of dialysis, and severe anemia requiring blood transfusion. PRES has been reported in other patients with COVID-19. Kishfy et al. reported PRES in two patients, both of whom had only moderate blood pressure fluctuations, both of whom had acute kidney injury, and one of whom had received toculizumab [5]. Parauda et al. reported four cases from one center: all had preceding renal injury and hypertension, one of whom had received tocilizumab, and two of whom had EEG-confirmed seizures [6]. Several of the previously reported cases had received hydroxychloroquine. The current patient had not received either tocilizumab or hydroxychloroquine, and developed PRES. A retrospective cohort study involving neuroimaging (CT and MRI) in patients with COVID-19 reported PRES in 1.1% [7].

The patient’s renal failure, blood transfusions with sickle cell disease, and focal seizures may also have played a combined role in the development of PRES. It is unclear if SC patients have a higher frequency of PRES predisposing factors than the general population or if SC is an independent risk factor for PRES [8], but seizures are the primary reason for SCD-associated hospitalization 12.5% of the time [9]. In addition, it is now known that SCD patients who become infected with SARS-CoV-2 have a high risk for a severe disease course and a high case-fatality rate [10].

It is unclear what fraction of patients with COVID-19 infection present with seizures. Asadi-Pooya et al. emphasized in a systematic review that patients with COVID-19 who had seizures early in the pandemic were not investigated thoroughly [11]. In a retrospective analysis of 32 patients who presented with COVID-19 infection and a negative history of epilepsy but who had an EEG, we found 11 with electrographic epileptiform discharges (8) or seizure activity (3), of which the current case is one [12].

## 4 Conclusions

COVID-19, either through its effect on the **angiotensin converting enzyme-2** receptors, endothelial injury, secondary effects on blood pressure and renal function, or due to the effects of medications used to treat it, may increase the risk of seizures in infected patients, with the development of PRES being one possible reason. The combination of COVID and sickle cell disease may raise the risk to develop PRES and could contribute to higher mortality rates from COVID-19 in patients with sickle cell disease.

## Ethical statement

The patient provided informed consent for publication of this case report.

## Declaration of Competing Interest

The authors declare that they have no known competing financial interests or personal relationships that could have appeared to influence the work reported in this paper.

## Footnotes

Appendix A Supplementary data to this article can be found online at https://doi.org/10.1016/j.ebr.2021.100436.
